# CXCR3 ameliorates neutrophil-dependent disease severity in SARS-CoV-2 infection by regulating CD4^+^ T cell recruitment

**DOI:** 10.3389/fimmu.2026.1823264

**Published:** 2026-06-11

**Authors:** Md Jashim Uddin, Claire Fleming, Nick R. Natale, Duncan Hart, Brett Moreau, Anthony Day, Judith Allen, William A. Petri

**Affiliations:** 1 Division of Infectious Diseases and International Health, Department of Medicine, University of Virginia Health System, Charlottesville, VA, United States; 2 Henry M Jackson Foundation for the Advancement of Military Medicine, Bethesda, VA, United States; 3 Department of Microbiology, Immunology and Cancer Biology, School of Medicine, University of Virginia, Charlottesville, VA, United States; 4 Neuroscience Graduate Program, University of Virginia, Charlottesville, VA, United States; 5 Lydia Becker Institute of Immunology and Inflammation, School of Biological Sciences, University of Manchester, Manchester Academic Health Sciences Centre, Manchester, United Kingdom; 6 Wellcome Trust Centre for Cell-Matrix Research, School of Biological Sciences, Faculty of Biology Medicine and Health, University of Manchester, Manchester Academic Health Sciences Centre, Manchester, United Kingdom; 7 Department of Pathology, University of Virginia School of Medicine, Charlottesville, VA, United States

**Keywords:** CD4+T cells, COVID-19, CXCR3, neutrophils, SARS-CoV-2

## Abstract

Understanding the host immune response to SARS-CoV-2 infection is critical for developing effective immunotherapeutic interventions. Using bulk RNA sequencing of lung tissue from mock-infected and mouse-adapted SARS-CoV-2 strain MA-10-infected mice, we identified CXCL9, CXCL10, and CXCL11 as among the most upregulated transcripts. Notably, their shared receptor, CXCR3, was also upregulated, suggesting activation of the CXCL9/10/11-CXCR3 axis in the lungs. Using spectral flow cytometry, we observed that the increased recruitment of CXCR3^+^ immune cells, particularly T cells, innate lymphoid cells (ILCs), and macrophages, correlated with milder disease outcome. Blocking CXCR3 signaling using monoclonal antibodies resulted in worsened disease, which was accompanied by reduced recruitment of T cells, ILCs, and macrophages, and a marked increase in neutrophil infiltration. Depletion of neutrophils using αLy6G antibodies in CXCR3-blocked mice alleviated disease severity, indicating that CXCR3 signaling mitigated neutrophil-driven pathology. CXCR3 blockade failed to exacerbate disease in RAG2^-/-^ mice, suggesting that CXCR3-mediated protection requires adaptive immune cells. Adoptive transfer of CD4^+^ T cells from wild type (WT), but not CXCR3^-/-^, mice conferred protection in RAG2^-/-^ mice. Together, our findings establish a protective role for CXCR3-recruited T cells blocking neutrophil infiltration in the lung, highlighting the mechanistic importance of the CXCL9/10/11-CXCR3 axis in protecting the lung from SARS-CoV-2 infection.

## 1 Introduction

Severe acute respiratory syndrome coronavirus 2 (SARS-CoV-2) is responsible for COVID-19 disease, which is primarily characterized by inflammation in the lower respiratory tract. SARS-CoV-2 infections have a range of manifestations in infected individuals, including asymptomatic infection to severe illness, and have accounted for more than 700 million cases and 7 million deaths worldwide (1). Several factors are known to be risk factors for severe disease during SARS-CoV-2 infection, including increased age, obesity, male sex, and co-morbidities such as hypertension, heart failure, cardiac arrhythmia, diabetes, kidney failure, and chronic pulmonary disease (2, 3). Despite the availability of several effective vaccines, COVID-19 continues to be a global health concern. In addition to the acute disease, the post-acute sequelae after SARS-CoV-2 infection are a persistent burden for global health (4). Hence, continued efforts to investigate immune pathogenesis and identify therapeutic interventions are needed.

Like many other infectious diseases, a balanced immune response at the site of infection is key to resolving SARS-CoV-2 infection, while a dysregulated immune response is responsible for severe pathology. The immune hyperresponsiveness resulting from the SARS-CoV-2 infection is frequently described as a “cytokine storm”. An overabundance of cytokines/chemokines, including IL-2, IL-6, IL-7, IL-10, IL-13, IFNg, IL-17a, GSCF, IP10, MCP1 (CCL2), MIP1A (CCL3), and TNF, have been described in human and mouse studies to be upregulated in lung and plasma samples following SARS-CoV-2 infection in severe cases (5–8). However, there is a knowledge gap of the role that these cytokines or chemokine are playing. It remains largely unknown whether the production of these proteins results in severe diseases or whether they are part of a protective host defense response. Cytokine-targeted therapies are being tested and found to be effective in treating COVID-19. For instance, a Janus kinase inhibitor, baricitinib, that targets intracellular signaling by different cytokines was found to be effective in providing faster recovery from COVID-19 (9). Similarly, our group previously identified that the type 2 cytokine IL-13 drives pathogenesis and its inhibition with monoclonal antibodies dampens COVID-19 disease severity (7, 10). Therefore, investigating the complex interaction of distinct immune responses and understanding the contribution of these immune mediators to SARS-CoV-2 infection and progression is crucial to designing effective immune therapeutics.

Neutrophils appear at sites of infection to limit the spread of pathogens through phagocytosis, secreting reactive oxygen species, or releasing neutrophil extracellular traps (NETs) (11, 12). However, a prolonged neutrophil response or dysregulated activation at the site of inflammation contributes to severe pathology (13). Recent studies revealed that induced expression of neutrophil adhesion factors correlates with impaired pulmonary circulation during SARS-CoV-2 infection (14). In addition, SARS-CoV-2 infection can promote the release of NETs, which can cause coagulation, and drive the differentiation of neutrophils to myeloid-derived suppressor cells, leading to the suppression of T cell activity (15, 16). Conversely, the recruitment of CD4^+^ FoxP3^+^ T cells in the lung during acute injury dampens neutrophil recruitment (17), suggesting that T cell and neutrophil responses counterbalance each other. There remain knowledge gaps about how the complex interactions between neutrophils and T cells shape COVID-19 pathology.

Signaling via chemokines and their receptors regulates the trafficking of immune cells at the site of inflammation. The interferon-regulated chemokines, CXCL9/10/11, signal through the CXCR3 receptor that is found in different immune cells, including T cells, NK cells, and macrophages (18, 19). CXCL10 is a common responder to different viral infections in the lung, including rhinovirus, respiratory syncytial virus, influenza, and SARS-CoV-2 (20–22). The role of CXCL9/10/11-CXCR3 signaling in COVID-19 pathogenesis has not been investigated.

Here we report studies in mice infected with the mouse-adapted strain of SARS-COV-2 (MA10) that demonstrate that upregulation of CXCL9/10/11-CXCR3 signaling is a protective host defense response. We found that inhibiting this signaling pathway using monoclonal antibodies against CXCR3 increased mortality and clinical scores in mice. We revealed that mice with milder disease following intranasal challenge with a mouse-adapted strain of SARS-CoV-2 had a higher number of CXCR3^+^ T cells, ILCs, and macrophages in broncho alveolar lavage fluid (BALF) compared to the mice with severe disease. CXCR3 signaling was crucial to the inhibition of neutrophil recruitment as blocking CXCR3 led to neutrophil infiltration. Additionally depleting neutrophils dampened disease severity. Utilizing RAG2^-/-^ mice, we demonstrated that CXCR3 signaling in lymphocytes was important in CXCR3-mediated protection. Finally, the adoptive transfer of CXCR3^+^CD4^+^T was sufficient to protect RAG2^-/-^ mice. In summary, CXCR3 signaling provided protection from SARS-CoV-2 pathogenesis by regulating CD4^+^ T cell and neutrophil recruitment to the lung.

## 2 Result

### 2.1 SARS-CoV-2 infection activates the CXCL9/10/11-CXCR3 pathway in murine lung

**Figure 1:**
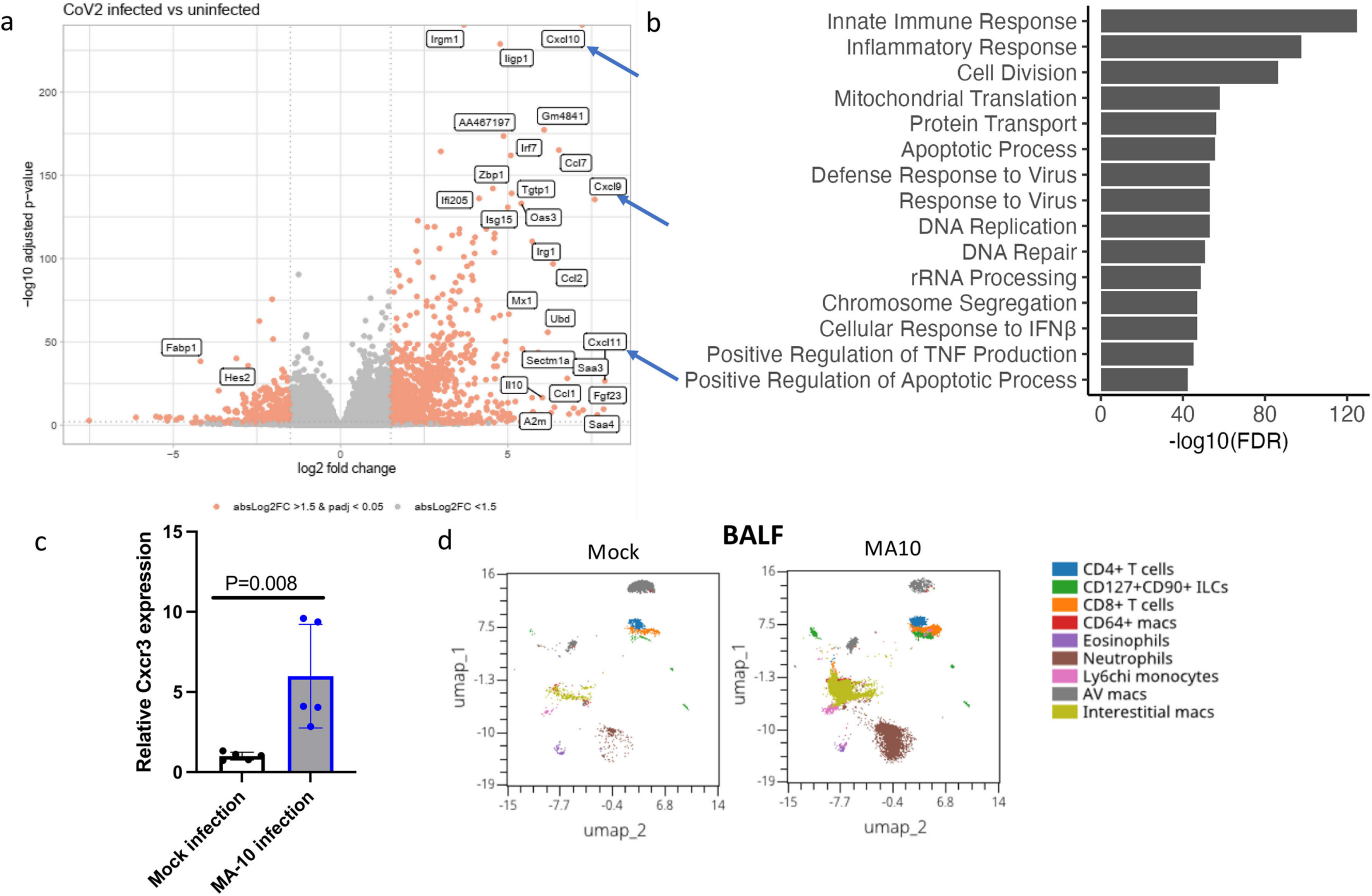
SARS-CoV-2 infection increases the expression of Cxcl9, Cxcl10, Cxcl11, and their receptor Cxcr3. 16-20-weeks-old C57BL/6J mice were infected with 1.5 x 104 PFU of mouse-adapted (MA-10) SARS-CoV-2 virus through intranasal inoculation. Bulk RNAseq was done on lung tissues to determine the differential gene expression. Spectral flow cytometry was conducted to assess immune infiltration in bronchoalveolar lavage fluid (BALF). **(a)** Differential gene expression between mock and MA-10 infected mice. **(b)** Pathway analysis using upregulated genes upon MA-10 infection. **(c)** Cxcr3 mRNA levels. **(d)** Immune phenotyping of BALF-isolated immune cells. **(a–c)** n=5 mice per group; **(d)** Mock, n = 4; MA-10, n = 6 mice. Immunophenotyping data shown are representative of two independent experiments. For **(c)**, data are mean values ± SEM; statistical significance was determined by unpaired *t*-test.

To identify host protective immune responses following SARS-CoV-2 infection, we analyzed transcriptional changes in mouse lungs following infection by a mouse-adapted strain of SARS-CoV-2 (MA-10) (23). Bulk RNAseq was performed on lung tissues collected at day 6 pi from mock-infected and MA-10-infected mice. Differential gene expression analysis between the mock-infected and the MA-10-infected lungs revealed that the chemokines Cxcl9, Cxcl10, and Cxcl11 were among the top upregulated genes after MA-10 infection (**Figure 1a**). To regulate the chemoattraction of immune cells, these three chemokines bind with a unique receptor, CXCR3 (18, 24, 25), which is also induced by MA-10 infection (**Figure 1c**). We performed DAVID Gene Ontology term enrichment analysis (26) to identify the potential functional significance of differentially expressed genes. Pathways involved in the innate immune response, and inflammation were among the top five enriched pathways (**Figure 1b**). The pathways involved in the ‘defense response to the virus’, and ‘response to virus’ were also among the top ten enriched pathways (**Figure 1b**). We were interested to know which gene sets contributed to the enrichment of these pathways and found that CXCL9 and CXCL10 were among the genes involved. Next, we performed spectral flow cytometry in order to measure the recruitment of innate and adaptive immune cells in the airway of mice after MA-10 infection. Bronchoalveolar lavage fluid (BALF)-isolated immune cells were stained to determine the abundance of eosinophils, neutrophils, monocytes, ILCs, alveolar macrophages, interstitial macrophages, CD4^+^ T cells, and CD8^+^ T cells. In BALF, there was a robust induction of all these immune cells except the alveolar macrophages, whose abundance decreased during MA-10 infection (**Figure 1d**; **Supplementary Figure 1**). Altogether, our data revealed that MA-10 infection activated the CXCL9/10/11-CXCR3 pathway, and this activation was associated with the recruitment to the alveoli of immune cells.

### 2.2 SARS-CoV-2 infection induces the recruitment of CXCR3^+^ cells

**Figure 2:**
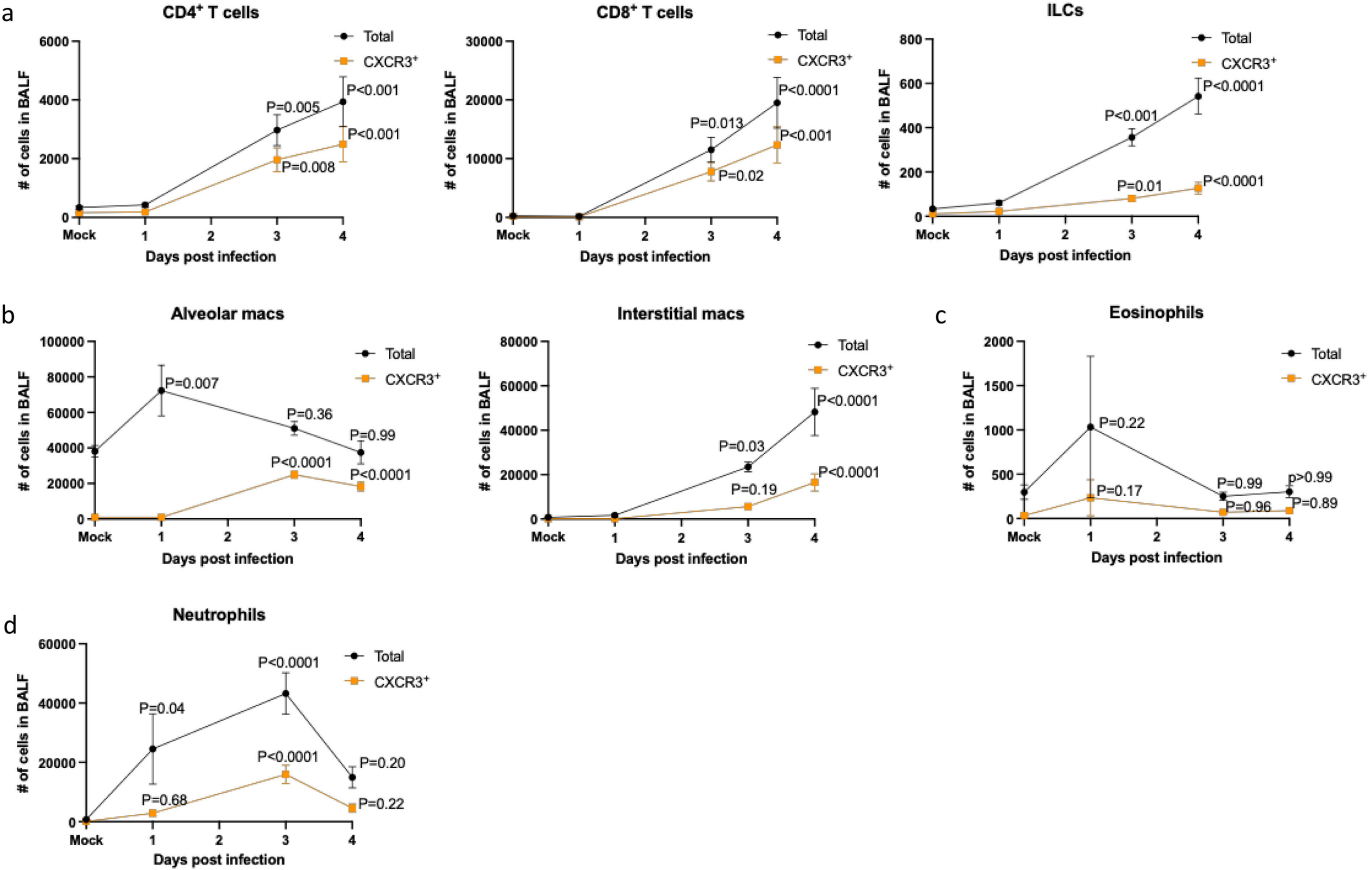
SARS-CoV-2 infection induces recruitment of CXCR3^+^ cells into the alveoli of mice. 16-20- week-old C57BL/6J mice were infected with the MA-10 virus through intranasal inoculation. Mice were harvested on days 1, 3, and 4 pi to collect BALF cells. Immunophenotyping was performed to determine total and CXCR3^+^ cells. **(a)** Counts of CD4^+^ T cells, CD8^+^ T cells, and ILCs. **(b)** Counts of alveolar macrophages, and interstitial macrophages. **(c)** Counts of eosinophils. **(d)** Counts of neutrophils. Mock, n = 8 mice; Day 1, n = 5 mice; Days 3, and 4, n=8 mice per time point. P-values at different time points were determined by comparison with mock infection. Statistical significance was determined by a one-way ANOVA test. Comparisons between multiple groups were performed using Dunnett’s test. Data are mean values ± SEM.

Next, we investigated the kinetics of immune cell infiltration in the airways of MA-10-infected mice. BALF cells were isolated from MA-10-infected mice on days 1, 3, and 4 post-challenge. On day 1 post challenge, we did not observe a noticeable change in total numbers of CD4^+^T cells, CD8^+^T cells, ILCs, or interstitial macrophages (**Figures 2a, b**). A continuous increase in these cell types was observed on days 3 and 4 (**Figures 2a, b**). Total counts of alveolar macrophages, and eosinophils increased in the airways on day 1 post-challenge (**Figures 2b, c**). On day 3 and day 4, alveolar macrophages and eosinophils decreased (**Figures 2b, c**). Total count of neutrophils increased until day 3, followed by a sharp decrease on day 4 (**Figure 2d**); despite this decline the count was still noticeably high compared to the mock-infected mice, suggesting a persistent inflammation in the infected lung. These data suggested that immune cell subsets respond differently and followed distinct kinetics after MA-10 infection. Since the increased expression of Cxcr3 and its ligands Cxcl9, Cxcl10, and Cxcl11 was associated with the higher number of immune cells in BALF (**Figure 1d**), we were interested to investigate the cellular sources of CXCR3 and if the abundance of CXCR3^+^ cells altered following MA-10 infections. A majority of the CD4^+^T cells and CD8^+^T cells were positive for CXCR3 (**Figure 2a**; **Supplementary Figure 2**). The kinetics of the recruitment of CXCR3^+^ cells followed a similar trend to that of the total cells (**Figure 2a**). In addition, infiltration of other CXCR3^+^ cells, including ILCs, macrophages, eosinophils, and neutrophils increased in the airways after the MA-10 infection (**Figures 2a–d**; **Supplementary Figure 2**). These results suggested that MA-10 infection regulates the trafficking of CXCR3^+^ immune cells.

### 2.3 Increased CXCR3^+^ immune cells are associated with the recovery following SARS-CoV-2 infection

**Figure 3:**
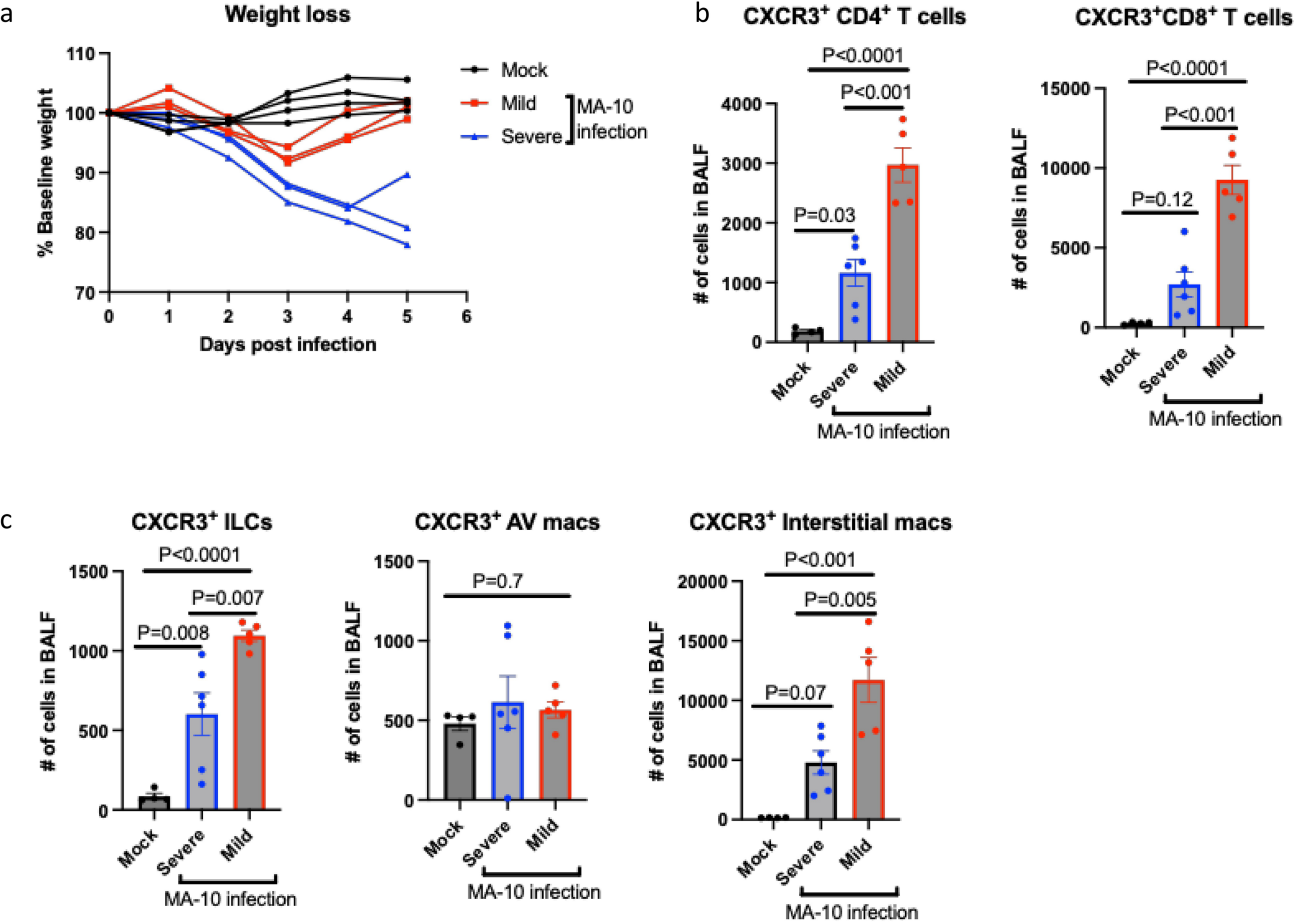
A higher number of CXCR3^+^ T cells, ILCs, and macrophages correlates with milder disease following SARS-CoV-2 infection. 16-20-week-old C57BL/6J mice were infected with the MA-10 virus through intranasal inoculation. Mice were harvested on day 5 pi to collect BALF cells. **(a)** Weight loss as an indicator of disease severity. **(b)** Total count of CXCR3^+^ T cells. **(c)** Total count of CXCR3^+^ ILCs, and macrophages. Mock, n=4 mice; Severe, n=6 mice; mild, n=5 mice. Data are combined from two independent experiments. Statistical significance was determined by a one-way ANOVA test. Comparisons between multiple groups were performed using Tukey’s test. Data are mean values ± SEM.

The clinical spectrum of SARS-CoV-2 infection is heterogeneous in humans and mice (27–29). We were interested in investigating whether the quantity of CXCR3^+^ cells was associated with infection outcomes. We compared the numbers of CXCR3^+^ immune cells between mock-infected, MA-10-infected mice that had severe disease, and MA-10-infected mice that had mild disease and recovered by day 5 of pi (**Figure 3a**). Mice that lost more than 15 percent of their baseline weight at any time during the course of infection and did not recover to >90% of their baseline weight by day 5 were considered to have severe disease. The mice with mild disease had significantly higher numbers of CXCR3^+^ T cells, ILCs, and interstitial macrophages compared to either mock-infected or MA-10-infected mice with severe disease (**Figures 3b, c**). Significant differences in CXCR3^+^ alveolar macrophages were not observed between the groups (**Figure 3c**). Our data revealed that a higher number of CXCR3^+^ cells, especially CD4^+^T cells, CD8^+^T cells, ILCs, and interstitial macrophages, were associated with the recovery following infection. Our findings identified a potential role of CXCR3^+^ immune cells in providing protection against SARS-CoV-2 infection.

### 2.4 CXCR3 signaling protects from SARS-CoV-2-associated disease severity

**Figure 4:**
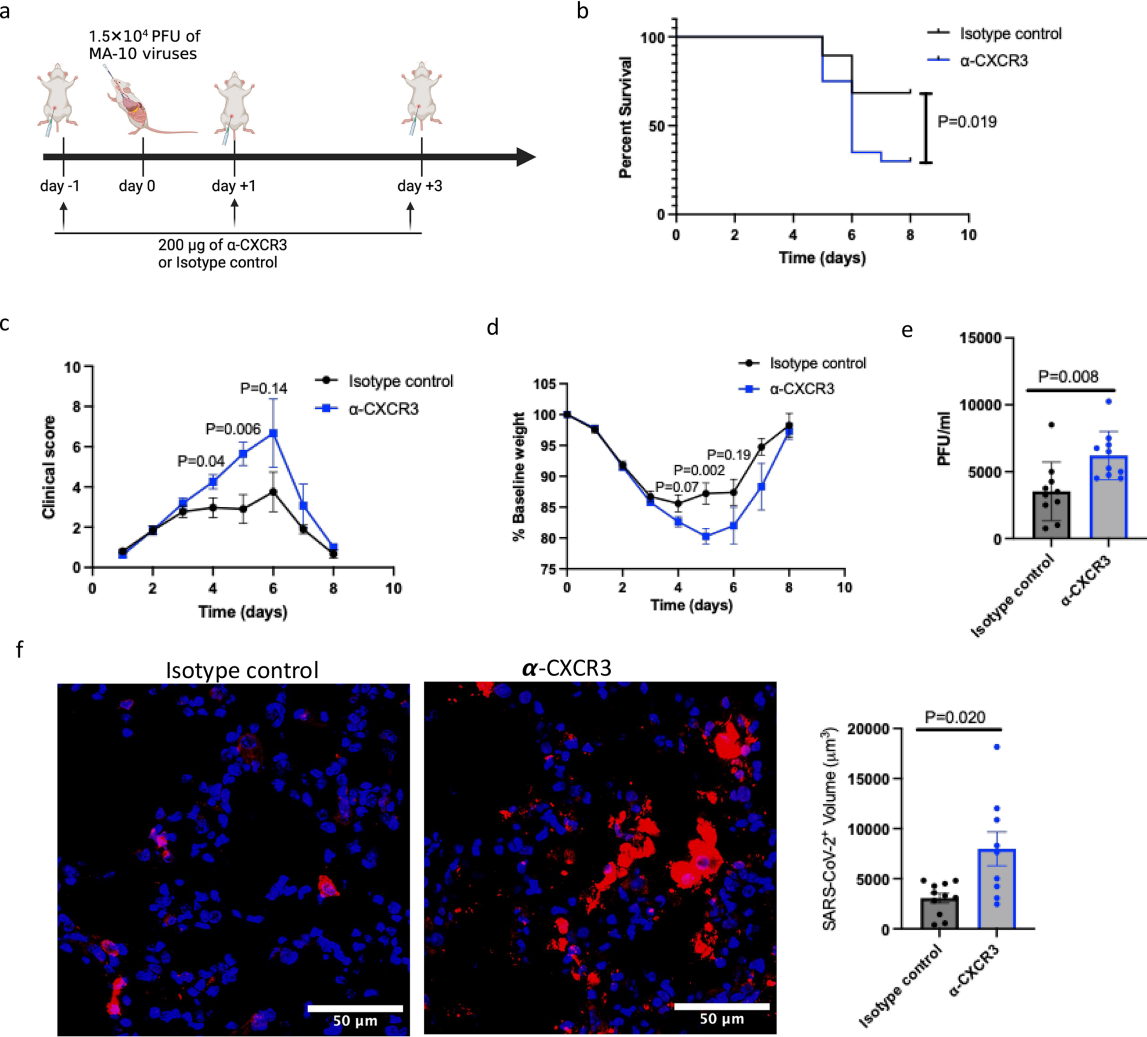
αCXCR3 treatment increases susceptibility to SARS-CoV-2 infection. Mice were treated with 200 üg of αCXCR3 antibody or isotype control on days -1, +1, and +3. All mice were challenged with MA-10 virus via the intra-nasal route. **(a)** Experimental design. **(b)** Survival curve. **(c)** Clinical score. **(d)** Weight loss. **(e)** Viral load in lungs measured by plaque assay on day 5 pi. **(f)** Immunostaining of lung sections shows the volume occupied by the SARS-CoV-2 nucleocapsid (red). **(b)** isotype control, n=19; α-CXCR3, n=20; **(c)** Isotype control, n=15, α-CXCR3, n=16 on day 0; **(d)** Isotype control, n=25, α-CXCR3, n=26 on day 0; **(e)** n=10 mice per group **(f)** Isotype control, n=11, α-CXCR3, n=9; Each symbol represents a value from an individual animal. Values are the mean of 4 field of views. Data are pooled from 2–3 independent experiments. The p-value for the survival curve was determined using the Log-rank (Mantel-Cox) test. P-values for clinical scores, and weight loss were determined by unpaired t-test. Data are mean values ± SEM. The experimental design was created using BioRender.com.

In several studies, CXCR3 signaling has been shown to be associated with both disease severity and recovery following SARS-CoV-2 infection (30–32). In severe COVID-19 patients, the abundance of CXCR3^+^ T cells was identified to be correlated with survival (30). However, the role of CXCR3 signaling has not been fully investigated. Since having a higher number of CXCR3^+^ immune cells was associated with milder disease and recovery, we hypothesized that CXCR3 signaling is protective against SARS-CoV-2 infection. To test this hypothesis, CXCR3 signaling was inhibited by treating mice intraperitoneally with 200μg of an αCXCR3 antibody on day -1, +1, and +3 of MA-10 challenge (**Figure 4a**). A statistically significant increase in mortality was observed compared to the isotype control-treated mice (**Figure 4b**). The αCXCR3 administered mice also had higher clinical scores and weight loss (**Figures 4c, d**). We were interested in determining whether CXCR3 signaling helped to clear viral load and thus provided protection. We quantified viral loads using a plaque assay from lung tissue lysates. The plaque assay showed that the αCXCR3-treated mice had a modest but statistically significant increase of viral loads measured in lung tissue lysates (**Figure 4d**). In addition, fluorescence staining of lung sections showed higher levels of SARS-CoV-2 nucleocapsid in αCXCR3-treated mice (**Figure 4f**). Therefore, our data demonstrated that CXCR3 signaling protected mice from MA-10-mediated disease severity.

### 2.5 Inhibiting CXCR3 signaling lowers the recruitment of Th cells, ILCs and macrophages, and increases neutrophils

**Figure 5:**
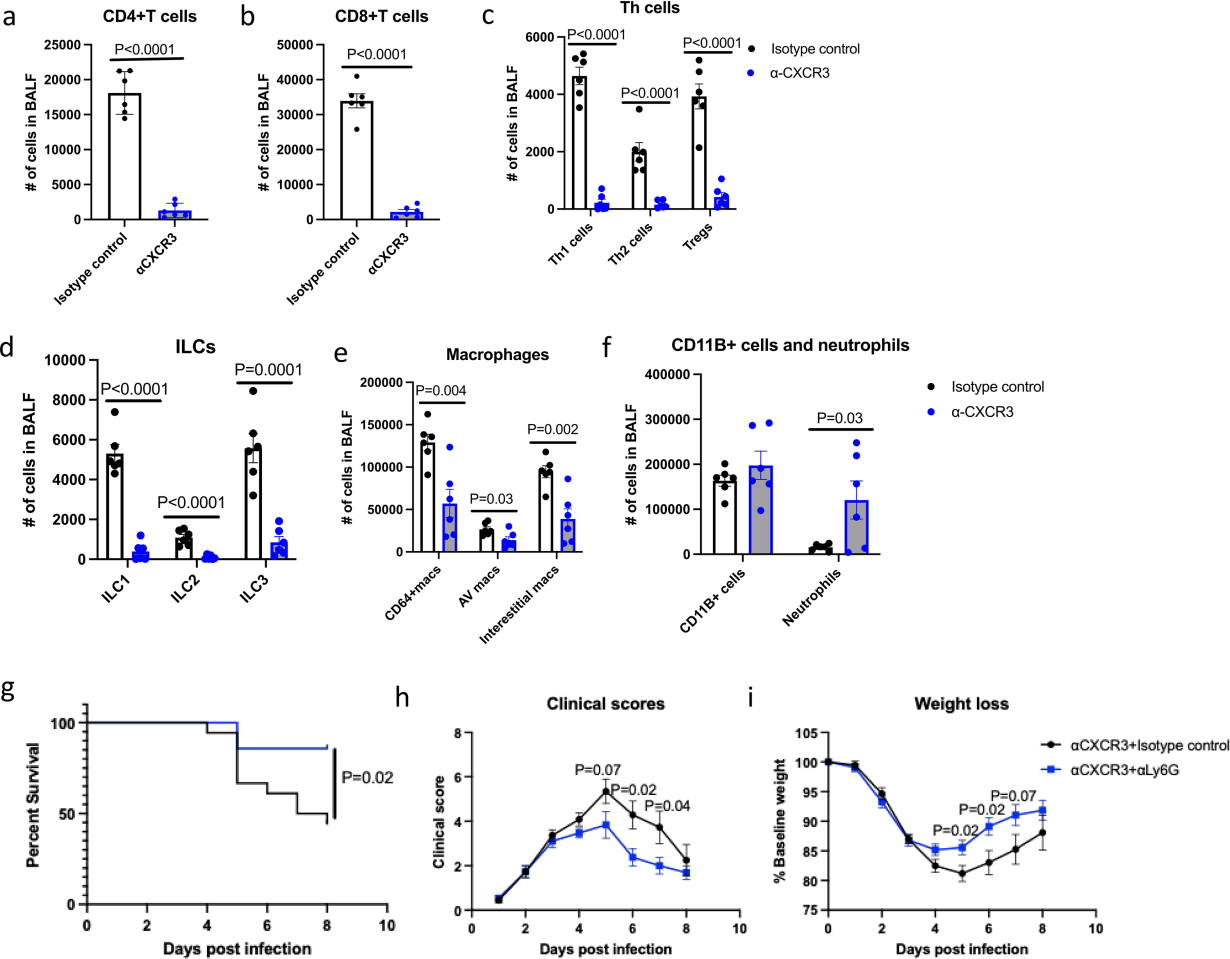
αCXCR3-mediated increased susceptibility to SARS-CoV-2 infection is neutrophil-dependent. **(a–f)** Mice were treated with 200 µg of αCXCR3 antibody or isotype control on days -1, +1, and +3. All mice were challenged with MA-10 virus via the intra-nasal route. On day 5 pi BALF was collected for immune phenotyping. Total cell numbers in BALF were measured for: **(a)** CD4^+^ T cells, **(b)** CD8^+^ T cells, **(c)** Th cells, **(d)** ILCs, **(e)** macrophages, and **(f)** CD11b^+^ cells and neutrophils. **(g–i)** For the neutrophil depletion, mice were treated with αLy6G or isotype control on days -1, +1, +3 and +5, in addition of administering αCXCR3 antibody to all mice. **(a–f)** n=6 mice in each group. **(g–i)** αCXCR3+isotype control, n=18; αCXCR3+ αLy6G, n=15. **(a–f)** Data are representative of two independent experiments. **(g–i)** Data are pooled from two independent experiments. Data are mean values ± SEM. P-value for the survival curve was calculated using the Log-rank (Mantel-Cox) test. P-values for immunophenotyping, clinical scores, and weight loss data were determined by unpaired t-test.

Since spectral flow cytometry showed that a wide range of immune cells express CXCR3 at steady-state and during MA-10 infection (**Figures 2a–c**), we were interested to investigate if αCXCR3-mediated susceptibility to MA-10 was associated with the alteration of immune cell recruitment in the airways. Supporting the findings that a majority of T cells expressed CXCR3 in BALF during MA-10 infection (**Figure 2a**), there was a robust reduction of T cells during αCXCR3 treatment (**Figures 5a, b**). We characterized CD4^+^T cell subsets and observed that Th1 (T-bet^+^), Th2 (Gata-3^+^), and Treg (Fox-P3^+^) cells declined significantly in the αCXCR3 treated mice (**Figure 5c**). We also observed a sharp reduction of ILCs in the αCXCR3-treated mice (**Figure 5d**). Although not as robust as the effect on T cells and ILCs, there was a significant decrease in total number of CD64^+^ macrophages, alveolar macrophages, and interstitial macrophages (**Figure 5e**). Several studies reported that a higher neutrophil-to-lymphocyte ratio predicted severe illness and mortality in COVID-19 patients (33–35). In accordance with that, we observed a significantly elevated level of neutrophils in BALF during αCXCR3 treatment (**Figure 5f**).

Dysregulated cytokine responses, aka “cytokine storm,” have been recognized to be associated with severe diseases during SARS-CoV-2 infection. We performed a multiplex Luminex assay on lung tissue lysates to determine if αCXCR3 treatment regulated cytokine responses. The type 2 cytokine IL-13 and type 3 cytokine IL-6 have been found to be associated with severe COVID-19, and inhibiting these cytokines improved disease outcomes (7, 36, 37). Although the IL-13 level was below the detection limit of the assay, another type-2 cytokine, IL-5, and type-3 cytokines, IL-6 and IL-22, were not significantly different between the groups (**Supplementary Figure 3**). Studies have identified that regulated cellular responses by the type-1 cytokine IFNγ were important in controlling viruses during primary infection and vaccine-mediated protection (38, 39). Interestingly, αCXCR3-treated mice had significantly lower levels of IFNγ and IL-27 in lung lysates (**Supplementary Figure 3**).

Thus, our data revealed that the αCXCR3-mediated increase in susceptibility to SARS-CoV-2 was associated with a decreased level of T cells, ILCs, macrophages, and an increased number of neutrophils. The data also suggest that the CXCR3-regulated protection was associated with IFNγ but independent of type-2 and type-3 cytokine responses.

### 2.6 αCXCR3-regulated susceptibility to SARS-CoV-2 infection is neutrophil-dependent

Persistent neutrophilia during SARS-CoV-2 infection is associated with critical illness and mortality (33–35). Dysregulated neutrophilic infiltration impairs pulmonary circulation and perpetuates the inflammatory responses by enhancing destruction of airway epithelial cells (14, 40). Since neutrophil clearance is impaired in the airways of αCXCR3-treated mice, we tested if neutrophils are responsible for increased disease severity in these mice. To test this, we administered αCXCR3 to mice as described above (**Figure 4a**). Neutrophils were depleted in αCXCR3 mice via i.p. injection of Ly6G antibody according to the previously published protocol (41). Interestingly, αLy6G-treated mice had reduced mortality compared to the isotype control group (**Figure 5g**). The αLy6G-administered group lost less weight and had significantly diminished clinical scores (**Figures 5h, i**). We concluded that CXCR3 signaling dampened neutrophilic infiltration, and that increased susceptibility to MA-10 infection during αCXCR3 treatment was dependent on neutrophil infiltration.

### 2.7 Adoptive transfer of WT CD4^+^ but not CXCR3^-/-^ CD4^+^ T cells is sufficient to protect RAG2^-/-^ mice

**Figure 6:**
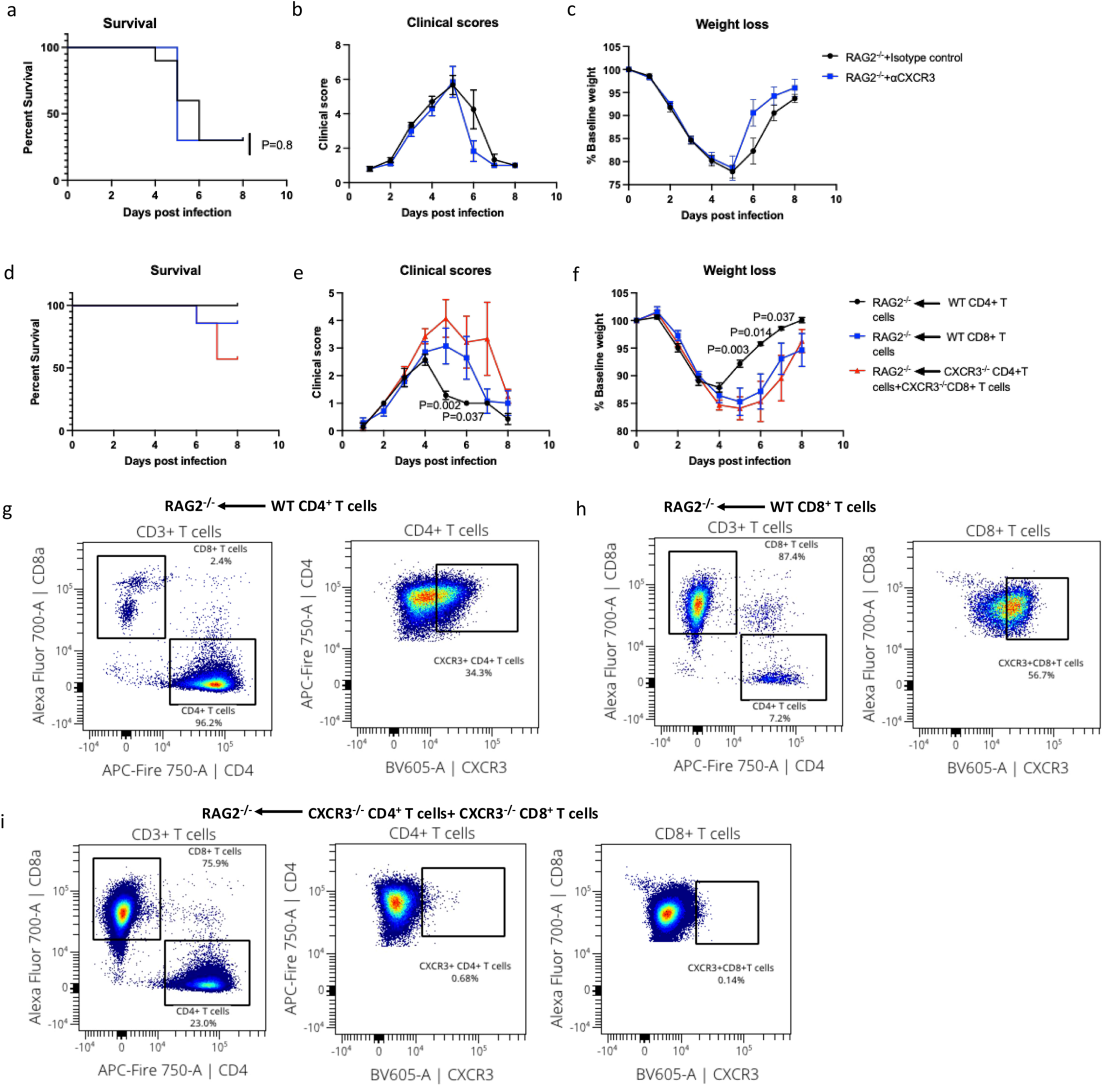
Adoptive transfer of WT CD4^+^ T cells is sufficient to protect RAG2^-/-^ mice. **(a–c)** RAG2^-/-^ mice were treated with 200 μg of αCXCR3 antibody or isotype control on days -1, +1, and +3. All mice were then challenged with MA-10 virus via the intranasal route. **(a)** Survival curve. **(b)** Clinical score. **(c)** Weight loss. **(d–i)** RAG2^-/-^ mice were adoptively transferred with 1 million WT CD4^+^ T cells, WT CD8^+^ T cells, or CXCR3^-/-^ CD4^+^ and CD8^+^ T cells via retro-orbital injection. All mice were then challenged with MA-10 virus via the intranasal route. **(d)** Survival curve. **(E)** Clinical score. **(f)** Weight loss. **(g–i)** Flow cytometry was performed on splenocytes to assess reconstitution of the adoptively transferred cells. **(a–c)** n=10 mice per group; **(d–f)** n=7 mice per group. **(a–c)** Data are pooled from two independent experiments. Data are mean values ± SEM.P-values for the survival curves were determined using Log-rank (Mantel-Cox) test. P-values for clinical scores, and weight loss were determined by unpaired t-test.

Since CXCR3 is expressed by both innate and adaptive immune cells (**Figure 2**) and αCXCR3 treatment depletes T cells, ILCs, and macrophages during the MA-10 infection (**Figure 5**), we investigated whether CXCR3 signaling in adaptive immune cells plays a role during SARS-CoV-2 infection. As the majority of CD4^+^ T cells and CD8^+^ T cells expressed CXCR3, as measured by flow cytometry (**Figure 2**), we hypothesized that CXCR3 signaling in T cells is crucial for conferring protection against SARS-CoV-2 infection. We treated RAG2^-/-^ mice (deficient in T and B cells) with an αCXCR3 antibody or an isotype control. Unlike WT mice, blocking CXCR3 signaling in RAG2^-/-^ mice did not alter the susceptibility (**Figures 6a–c**), indicating that CXCR3 signaling in the adaptive immune response is critical. We flow sorted CD4^+^ T cells and CD8^+^ T cells from WT vs CXCR3^-/-^ mice and adoptively transferred them into RAG2^-/-^ mice. The ‘recipient’ mice received WT CD4^+^ T cells, or WT CD8^+^ T cells, or CXCR3^-/-^ CD4^+^ T and CD8^+^ T cells. After the adoptive transfer, mice were left to rest for two weeks, which allowed for reconstitution and differentiation of the transferred cells. Mice were then challenged with the MA-10 virus. In comparison to mice receiving CXCR3^-/-^ cells or WT CD8^+^ T cells, mice reconstituted with WT CD4^+^ T cells were protected from the MA-10 infection (**Figures 6d–f**). Reconstitution of the transferred cells was confirmed by flow cytometry on splenocytes (**Figures 6g–i**). We concluded that CXCR3 signaling in CD4^+^T cells was critical to provide protection from the SARS-CoV-2 infection.

## 3 Discussion

A major discovery of this work is that CXCR3 signaling is protective against SARS-CoV-2 infection. Several studies have demonstrated the expression of CXCR3 by heterogeneous cell types, including airway epithelial cells, CD4^+^ T cells, CD8^+^ T cells, NK cells, monocytes, and macrophages (29, 42–44). For example, the surface expression of CXCR3 in T cells regulates diverse functions, including their differentiation to effector or memory cells and migration of cells to the site of infection (45–47). During influenza infection, the recruitment of antigen specific CD4 ^+^T cells is dependent on CXCR3 surface expression (45). Likewise, in our study, a diverse population of CXCR3^+^ innate and adaptive immune cells migrated to alveoli during MA-10 infection (**Figure 2**). Interestingly, mice with milder disease had increased numbers of CXCR3^+^ T cells compared to the mice with severe disease. Therefore, the increased levels of CXCR3^+^ T cells might be contributing to recovery by dampening neutrophilic infiltration. In support of this, we observed higher neutrophilic infiltration in lungs when CXCR3 signaling was inhibited by antibody treatment. Moreover, interfering with neutrophilic infiltration prevented mice from having severe disease.

The interaction between neutrophils and T cells during SARS-CoV-2 infection is not fully understood. A recent study demonstrated the presence of polymorphonuclear myeloid-derived suppressor cells (PMN-MDSCs) in the PBMCs of severely infected patients (15). PMN-MDSCs, or inflammatory neutrophils, have the potential to suppress T cell proliferation and cytokine production (15, 48). In contrast, T cells, particularly regulatory T cells, contribute to tissue healing and dampening inflammation by limiting neutrophil responses at the site of infection (49, 50). During αCXCR3 treatment, we observed that different subsets of CD4^+^ T cells, including FoxP3^+^ CD4^+^ T cells, were drastically reduced in the alveoli of mice (**Figure 5c**). The reduction of FoxP3^+^ CD4^+^ T cells might be responsible for compromised neutrophil clearance and increased disease severity observed in the αCXCR3-treated mice (**Figure 5f**). While our data suggests the main effect of blocking CXCR3 was on T cell recruitment, it is important to note that CXCR3 surface expression can also regulate T cell functionality (51, 52).

The role of T cells in mitigating COVID-19 severity has been previously indicated by human studies, as critically ill patients had low lymphocyte-to-neutrophil and low lymphocyte-to-monocyte ratios (33, 35). The abundance of CXCR3^+^ CD8^+^ polyfunctional T cells in the circulation was also associated with milder disease (30). However, before our work there was no direct evidence that T cells-particularly CXCR3^+^ T cells-play a role in alleviating SARS-CoV-2 pathology. By performing adoptive transfer of T cells into RAG2^-/-^ mice, we provided evidence that CXCR3^+^ CD4^+^ T cells are sufficient to ameliorate disease severity after MA-10 infection.

During acute infection, lung-infiltrating effector T cells exert antiviral and anti-inflammatory responses by producing effector cytokines, including IL-10 and IFN-γ (53). IFN-γ treatment controls the replication of SARS-CoV-2 in human pulmonary epithelial cells by generating an antiviral nitric oxide (NO) response (39). In our MA-10 infection model, αCXCR3-mediated susceptibility was associated with a significant reduction of IFNγ in lung tissue lysates. However, we do not have sufficient data to determine whether IFN-γ plays a role in our model. Our observed phenotype could also be independent of IFN-γ levels in the lungs. We also observed a significant reduction in IL-27 levels in lung tissue lysates after αCXCR3 treatment. IL-27, released by antigen-presenting cells, has the potential to regulate T-bet^+^ Th1 cells (54). A recent study reported that IL-27 can also be secreted by regulatory T cells, which can further limit the inflammatory response (55). During influenza infection, IL-27 was observed to limit neutrophilic inflammation in the lungs (56). The induced neutrophil infiltration in MA-10-infected mice after αCXCR3 treatment could be due to decreased secretion of IL-27 by regulatory T cells. Exploring the contribution of IL-27 in limiting lung inflammation during SARS-CoV-2 infection could provide a novel therapeutic avenue.

Blocking CXCR3 signaling drastically reduced the recruitment of CD4^+^ T cells and CD8^+^ T cells, while the number of neutrophils in the airways increased. This suggests that the recruitment of most neutrophils is not mediated by CXCR3. Impaired recruitment of protective CXCR3^+^ immune cells might indirectly contribute to the increased neutrophil recruitment. Additionally, higher viral loads in anti-CXCR3-treated mice may also contribute to increased neutrophil infiltration.

A recent study by Majumdar et al., utilizing CXCL10^-^/^-^ mice, found that CXCL10 is protective against SARS-CoV-2 pathogenesis (57). The authors also observed that the compromised recruitment of CD4^+^ and CD8^+^ T cells in the lung parenchyma of CXCL10^-^/^-^ mice was associated with increased mortality. Supporting their findings, our data show that blocking the CXCL10 receptor, CXCR3, increases SARS-CoV-2 pathogenesis. Most importantly, our data provide evidence that CXCR3^+^ CD4^+^ T cells are protective. In addition, our findings provide mechanistic insight into how the interplay between CXCR3 signaling and neutrophils is crucial for lessening lung inflammation.

As we move past the pandemic, COVID-19 remains a global health concern due to the emergence of variants and sub-variants, along with waning immune responses after primary infection and vaccination. In addition, post-acute sequelae, or long-haul COVID, continues to be a public health burden. Continued investigation into host immune mechanisms is critical for the development of new therapeutic strategies. In this study, we identify a previously unrecognized role for CXCR3 signaling in protection against SARS-CoV-2 infection. Mechanistically, we show that blocking CXCR3 signaling increases neutrophil recruitment in the alveoli, contributing to disease severity. Neutrophil depletion ameliorates the effects of CXCR3 blockade, highlighting the importance of CXCR3 in regulating neutrophil-driven pathology. Furthermore, adoptive transfer of CD4^+^ T cells from CXCR3-sufficient mice demonstrates that CXCR3^+^ CD4^+^ T cells contribute to host defense. Our findings provide novel insight into how these cells contribute to the resolution of lung inflammation. We believe our study may have broader implications for understanding other forms of lung inflammation and suggest that therapeutic modulation of the CXCR3 axis may be a promising strategy for treating pulmonary disease.

## 4 Methods

### 4.1 Mice

Experiments were performed using 16- to 24-week-old, age-matched C57BL/6, RAG2^-/-^, and CXCR3^-/-^ mice. Mice were purchased from Jackson Laboratory and housed in specific pathogen- and opportunistic-free animal rooms. Upon arrival, all mice were maintained under specific pathogen-free conditions in the vivarium of the University of Virginia. All animal experiments were approved by the University of Virginia Institutional Animal Care and Use Committee.

### 4.2 Virus culture and propagation

MA-10 SARS-CoV-2 (BEI: NR-55329) was obtained from the Biodefense and Emerging Infections Research Resources Repository, National Institute of Allergy and Infectious Diseases (NIAID), National Institutes of Health (NIH). The parental SARS-CoV-2 MA virus was generated by genetically engineering the WT virus through the introduction of Q498Y/P499T substitutions into the spike protein (23). The MA-10 SARS-CoV-2 was generated via serial *in vivo* passaging of the parental SARS-CoV-2 MA virus, which increased the virulence of the strain (58). Upon receipt, the virus was propagated at the BSL-3 facility of the University of Virginia using a previously described protocol (7). Briefly, the virus was propagated in VERO E6 cells to generate working stock. In T75 flasks, 90% confluent VERO E6 cells were infected with SARS-CoV-2 MA-10 in serum-free DMEM. VERO E6 cells and virus were incubated for 2 hours at 37 °C with 5% (v/v) CO_2_. The medium was then removed and replaced with DMEM supplemented with 10% (w/v) FBS. After 2 days of incubation, detachment of the VERO cells was observed in the flask, indicating the cytopathic effects of the virus. The cell supernatant was collected, filtered through a 0.22 μm filter (Millipore, SLGP003RS), and centrifuged at 300 × g for ten minutes at 4 °C. The supernatant containing virus was stored at –80 °C and later used to challenge mice.

RNA genomes of SARS-CoV-2 MA-10 stocks from the second passage were purified with an EZ1 DSP Virus Kit (Qiagen, 62724). Library preparation was performed with a cDNA-PCR Sequencing V14 kit (Oxford Nanopore Technologies, SQK-PCS114), and the cDNA output was sequenced using an Oxford Nanopore MinION. Consensus genome sequences were analyzed with Geneious Prime software (Dotmatics, v.2024.0.5) and demonstrated >99% pairwise sequence alignment with the reference genome for SARS-CoV-2 MA10 [NCBI GenBank accession number MT952602.1].

### 4.3 Challenge

Mice were challenged with 1–5 × 10^4^ PFU of MA-10 virus in 50 μl of saline via the intranasal route under 100 μl ketamine/xylazine sedation. Mice were monitored daily to record clinical scores and weight loss. Total clinical scores were calculated by combining weight loss (scores 0–5), activity (scores 0–3), and fur appearance and posture (scores 0–2).

### 4.4 αCXCR3 treatment

To inhibit CXCR3 signaling, mice were administered 200 μg of αCXCR3 monoclonal antibody (Bio X Cell, catalog #BE0249) or an isotype control antibody (Bio X Cell, catalog #BE0091) in 100 μl of PBS intraperitoneally on days –1, +1, and +3 of viral challenge.

### 4.5 Depletion of neutrophils

To deplete neutrophils we followed a previously published protocol (41). Each mouse was administered 100 μg of anti-rat Kappa immunoglobulin (Bio X Cell, MAR18.5, catalog #BE0122) intraperitoneally for two days prior to infection. Additionally, mice received 50 μg of anti-Ly6G (Bio X Cell, 1A8, catalog #BE0075-1) or isotype control (Bio X Cell, 2A3, catalog #BE0089) intraperitoneally on days -1, +1, +3, and +5 of challenge.

### 4.6 BALF collection and flow cytometry

To collect bronchoalveolar lavage fluid (BALF) cells, the alveoli were flushed three times with 1 ml of PBS. BALF was then centrifuged at 500 x for 5 minutes, and pelleted cells were plated and stained in 96-well plates. For live/dead staining, cells were incubated for 15 minutes at room temperature with 1 μl of Zombie NIR (BioLegend, 423105) or Live/Dead Blue (Thermo Scientific, L23105) dye in 100 μl of PBS. After incubation, cells were washed with FACS buffer (2% FBS in PBS) and subsequently surface stained with antibodies against LY6C (Alexa Fluor 488, BioLegend, 128022), LY6G (BV650, BioLegend, 127641), Siglec-F (PE, BioLegend, 155506), CD45 (Spark Violet 538, BioLegend, 103180), CD8a (AF700, BioLegend, 100730), CD4 (APC-Fire 750, BioLegend, 100460), CD11c (PE-Cy7, BioLegend, 117318), CD11b (BV480, Fisher Scientific, 566117), CD3 (APC, BioLegend, 100235), TCRβ (BV570, BioLegend, 109231), CD90.2 (BV785, BioLegend, 105331), CD127 (PE-Cy5, BioLegend, 135016), CXCR3 (BV605, BioLegend, 126523), and CD64 (BV421, BioLegend, 139309). For intracellular staining, cells were fixed and permeabilized using the FoxP3/Transcription Factor Staining Buffer Set (eBioscience, 00-5523-00). Cells were then stained for T-bet (PE/Dazzle 594, BioLegend, 644828), GATA3 (BV711, BD, 565449), RORγT (PerCP eFluor 710, eBioscience, 46-6981-82), RORγT (APC, eBioscience, 17-6981-82), and FoxP3 (PerCP eFluor 710, eBioscience, 46-5773-82) antibodies. Samples were assessed using a Cytek Aurora Borealis at the University of Virginia Flow Cytometry Core.

### 4.7 Viral titer

Viral titers were determined from lung tissue lysates using a plaque assay. Briefly, the left lung lobe from each mouse was dissected and stored in 1 ml of serum-free DMEM. Tissue lysates were prepared using a disposable tissue grinder. Lysates were centrifuged at 300 × g for 10 minutes, and the supernatants were collected and stored at –80 °C until plaque assay performance. Plaque assays were performed to determine viral titers. Briefly, VERO E6 cells were cultured in 6-well plates to 90% confluency. Cells were incubated with serially diluted lung tissue lysates at 37 °C with 5% CO_2_ for 2 hours to allow viral infection. Wells were then washed with PBS to remove unbound virus. Plates were overlaid with DMEM containing 2.5% FBS and 1.2% Avicel PH-101 (Sigma-Aldrich) and incubated for 48 hours. After incubation, the overlay was removed, and cells were fixed with 10% formaldehyde followed by staining with 0.1% crystal violet to visualize plaques. Plaques were counted and used to calculate viral titers.

Samples were tested at five different dilutions: 10¹, 10², 10³, 10^4^, and 10^5^. All samples were run in duplicate. Plaque numbers from the duplicate wells were averaged to calculate the viral titers. A 200-µL sample was inoculated into each well. For viral titer calculations, we used the data from the 10² dilution. We selected this dilution because at lower dilutions the plaque numbers could not be counted reliably, while at higher dilutions some samples were too dilute to produce any plaques. Viral titers were determined using the formula PFU/mL = (plaques × dilution × 5).

### 4.8 Immunofluorescence staining

For the immunostaining, lungs were drop-fixed in 10% formalin (Fisher Scientific) for 24 h at 4°C and transferred to 70% ethanol (Fisher Scientific) for 48 h at 4°C. Lungs were paraffin embedded, sectioned at 5 μm in thickness, and adhered to Superfrost Plus slides (Fisher Scientific) by the University of Virginia Research Histology Core. Slides were submerged in CitriSolv (Fisher Scientific) and systematically washed with ethanol and 1X PBS to remove paraffin. For protein antigen retrieval, slides were heated in a 10mM Sodium Citrate solution (Thermo Fisher Scientific) by the University of Virginia Biorepository Tissue Research Histology Facility. Slides were stored in 1X PBS (Thermo Fisher Scientific) at 4° C prior to deparaffinization.

The lung sections on slides were blocked for 1 h at room temperature (RT) with a blocking solution consisting of 2% donkey serum (Sigma-Aldrich), 1% BSA (Thermo Fisher Scientific), 0.1% Triton-X (Sigma-Aldrich), 0.05% Tween20 (Sigma-Aldrich) in 1X PBS (Thermo Fisher Scientific). Following blocking, slides were incubated for 14 h at 4°C with primary antibody master mix diluted in the blocking solution. Samples were stained with anti-SARS-CoV-2 Nucleocapsid (Genetex, 1:1000) for SARS-CoV-2 detection. Images of the samples were acquired by using Leica Application Suite X software (Leica Microsystems) to control a Leica Stellaris 5 Confocal Microscope (Leica Microsystems). Analysis of images was conducted using Imaris software (10.0.0).

### 4.9 Bulk RNA sequencing and pathway enrichment analysis

For RNAseq, the lower right lung lobe of each mouse was stored in TRIzol. RNA was extracted from lung tissue using a previously published protocol from our lab (7). RNA quality was assessed using the Agilent TapeStation RNA kit. Library preparation, sequencing, quality control, and read mapping were performed by the University of Virginia Genome Analysis and Technology Core (RRID: SCR_018883) (7). Once libraries were prepared and passed QC, sequencing was performed on the Illumina NextSeq 500 using a 150-cycle high output kit (400 million reads, 2 × 75 bp paired-end).

Downstream analysis of mapped gene counts was performed in R (version 4.4.1). Differential gene expression was calculated using the DESeq2 package (59), which excluded genes with low counts, normalized the data, estimated dispersions, and fit counts using a negative binomial model. Pathway enrichment analysis was performed using the Database for Annotation, Visualization, and Integrated Discovery (DAVID) Functional Annotation Tool. Briefly, a list of significantly upregulated genes was examined for enrichment using the Gene Ontology: Biological Processes Direct gene set. Significant gene sets were defined as those with an FDR-adjusted p-value < 0.05.

### 4.10 Adoptive transfer of T cells

For the adoptive transfer experiments, naïve CD44-negative CD4^+^ T cells and CD8^+^ T cells were flow-sorted from WT and CXCR3^-/-^ mice. After sorting, cells were washed with PBS and centrifuged at 500 × g for 5 minutes. Cells were then resuspended in sterile PBS at a concentration of 1 × 10^7 cells/ml. Cells were adoptively transferred into recipient RAG2^-/-^ mice via retro-orbital injection. Recipient mice received 1 million WT CD4^+^ T cells, or WT CD8^+^ T cells, or CXCR3^-/-^ CD4^+^ T cells and CD8^+^ T cells in 100 µL PBS. Mice were allowed to rest for a couple of weeks before being challenged with MA-10 viruses.

### 4.11 Cytokine analysis

Cytokines were measured from lung tissue lysates using a multiplex Luminex assay.

### 4.12 Statistical analysis

The p-value for survival curves was determined using the log-rank (Mantel–Cox) test. P-values for clinical scores and weight loss were determined using a nonparametric t-test. ANOVA was used to compare multiple groups. All analyses were performed using GraphPad Prism software.

## Data Availability

The datasets presented in this study can be found in online repositories. The names of the repository/repositories and accession number(s) can be found below: https://www.ncbi.nlm.nih.gov/geo/, GSE309229.
