# Supplementary material for: CXCR3 ameliorates neutrophil-dependent disease severity in SARS-CoV-2 infection by regulating CD4+ T cell recruitment
Source: Front Immunol. 2026 Jun 11;17:1823264. doi: 10.3389/fimmu.2026.1823264 (PMC13294051; doi:10.3389/fimmu.2026.1823264)
Supplement: Supplementary file 1 [file Table1.docx]

Supplementary Material

## Supplementary Figures

**Supplementary Figure 1.** (a) Flow cytometry gating strategy to immunophenotype BALF-isolated cells. (b) Total number of cells on day 5 in BALF during mock and MA-10 infection. Mock infection, n=4; MA-10 infection n=6. Data were analyzed using unpaired Welch’s t-tests. Multiple comparisons were corrected using the Benjamini–Krieger–Yekutieli false discovery rate method.

**Supplementary Figure 2.** **Flow cytometry gating strategy to determine CXCR3^+^ cells.**

16–20-week-old C57BL/6J mice were infected with MA-10 virus via intranasal inoculation. Flow cytometry was performed on BALF-derived cells. Gating strategies for CXCR3⁺ CD4⁺ T cells, CD8⁺ T cells, ILCs, eosinophils, neutrophils, and macrophages are shown. n=4-6 mice.


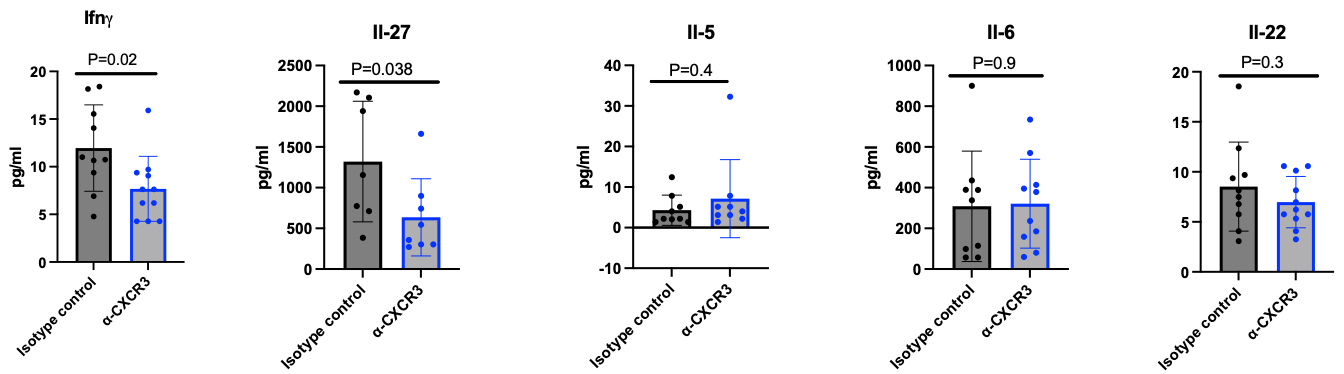


**Supplementary Figure 3.** **αCXCR3 treatment decreases type 1 cytokines IFNγ and IL-27:** A multiplex Luminex assay was done to determine the Typ1, Type2, and Type 3 cytokines in lung tissue lysates between isotype control and αCXCR3 treated mice. N=7-11 mice in each group. P-values were determined by Mann-Whitney test. Data are mean values ± SEM
